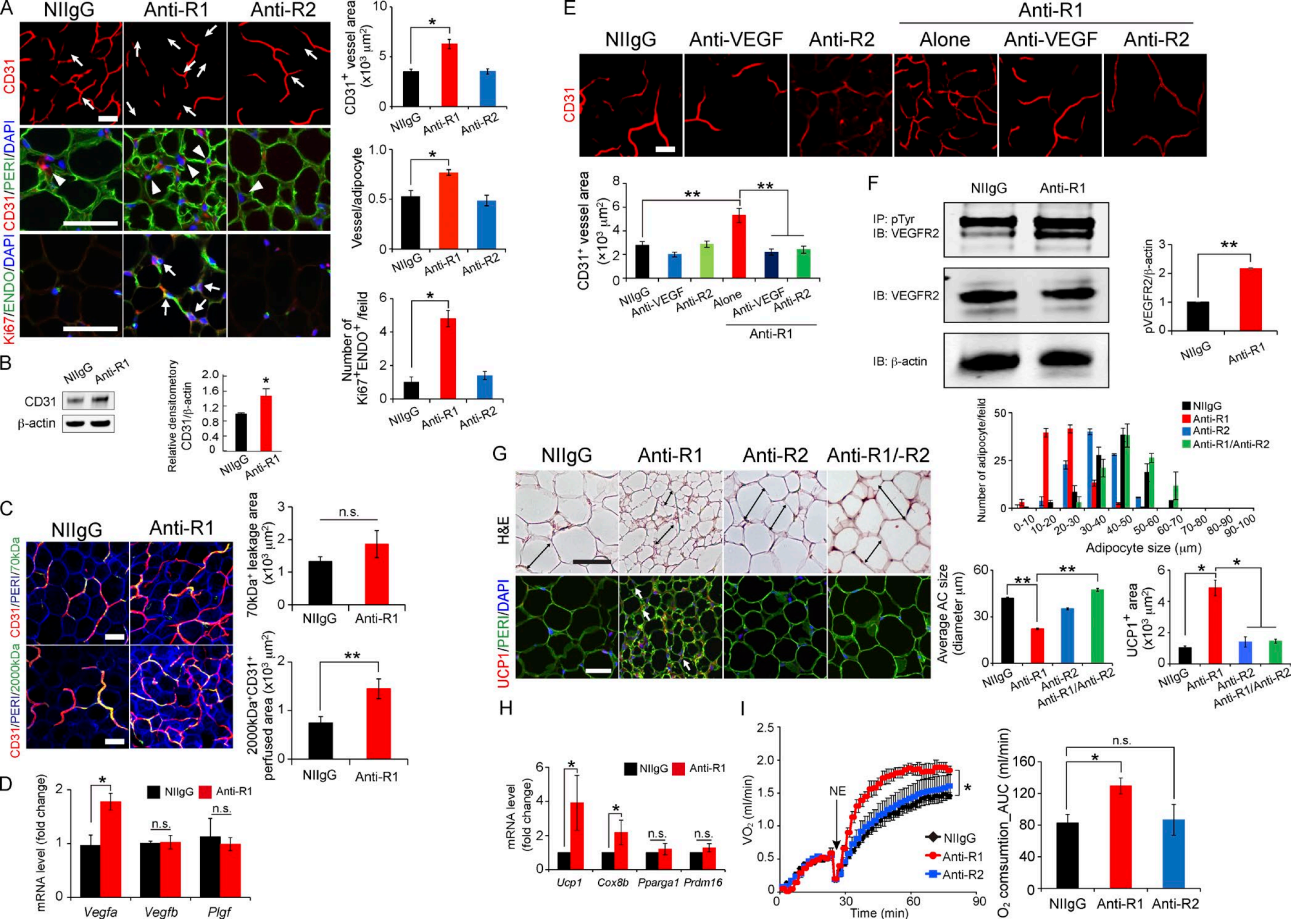# Correction: Ablation of endothelial VEGFR1 improves metabolic dysfunction by inducing adipose tissue browning

**DOI:** 10.1084/jem.2017101208112020c

**Published:** 2020-08-14

**Authors:** Takahiro Seki, Kayoko Hosaka, Carina Fischer, Sharon Lim, Patrik Andersson, Mitsuhiko Abe, Hideki Iwamoto, Yanyan Gao, Xinsheng Wang, Guo-Hua Fong, Yihai Cao

Vol. 215, No. 2 | 10.1084/jem.20171012 | January 5, 2018

The authors regret that the original version of Figure 1 contained incorrect CD31 images for anti-VEGFR2. The corrected figure is shown here. The error appears in print and in PDFs downloaded before August 7, 2020.

**Figure 1:**